# Parental Information-Use Strategies in a Digital Parenting Environment and Their Associations With Parental Social Support and Self-Efficacy: Cross-Sectional Study

**DOI:** 10.2196/58757

**Published:** 2024-12-19

**Authors:** Ryuta Onishi

**Affiliations:** 1 Faculty of Nursing Toyama Prefectural University Toyama-shi, Toyama-ken Japan; 2 Faculty of Health Sciences Hokkaido University Sapporo Japan

**Keywords:** parenting, information use, digital society, online information, social support, self-efficacy, parents, surveys, information seeking, information behaviors, resources, children, youth, pediatric

## Abstract

**Background:**

In today’s digital society, the acquisition of parenting information through online platforms such as social networking sites (SNSs) has become widespread. Amid the mix of online and offline information sources, there is a need to discover effective information-seeking methods for solving parenting problems.

**Objective:**

This study aimed to identify patterns of information use among parents of young children in the digital age and elucidate the characteristics of these patterns through a comparative analysis of parental social support and self-efficacy.

**Methods:**

An internet-based survey was administered to fathers and mothers of children aged 0-3 years. Convenience sampling, facilitated by an internet-based survey company, was adopted, and data from 227 fathers and 206 mothers were analyzed. The survey included questions on personal characteristics, frequency of use of different sources of parenting information (websites, SNSs, parenting apps, family, friends, and professionals), availability of parental social support, and parental self-efficacy. The Partitioning Around Medoids (PAM) clustering algorithm was used to identify patterns in parenting information use.

**Results:**

A total of 4 clusters were identified: multisource gatherers (n=161), offline-centric gatherers (n=105), online-centric gatherers (n=86), and minimal information gatherers (n=68). The availability of parental social support was perceived to be relatively higher among multisource and offline-centric gatherers compared with online-centric and minimal information gatherers. Parental self-efficacy was highest among multisource gatherers, followed by offline-centric and online-centric gatherers, and lowest among minimal information gatherers.

**Conclusions:**

This study contributes to the evidence that online information can effectively complement offline information in addressing parenting challenges, although its ability to fully replace offline sources remains limited. Parenting support professionals are encouraged to understand parents’ current information use strategies and actively foster their social relationships, helping them to adopt more diverse and comprehensive approaches to information use.

## Introduction

In the current digital age, the proliferation of applications designed to make pregnancy and parenting more convenient is changing the landscape of parenting [1,2]. The space for sharing parenting information and support has expanded from offline to online, becoming a normalized aspect of daily life [3]. The expansion of the online information space has provided many benefits for parents. Parents now have immediate access to a wide range of parenting information, including insights that are not available offline [4,5], and barriers to professional advice have reduced owing to parenting applications and online platforms [4]. Furthermore, online peer support has become increasingly active through platforms such as social networking sites (SNSs) [4], helping parents solve problems and learn about and establish parenting styles.

Since the outbreak of the COVID-19 pandemic, online family support services have rapidly expanded [6]. These services include professional family support through video conferencing tools [7] and online parenting programs [8]. The effectiveness of online parenting support programs has been shown to be comparable to that of in-person support programs [9], indicating that the digital society has brought about innovative advancements in the methodology of professional parenting support.

However, the online information space also presents certain challenges. Internet use that results in an information overload can overwhelm parents, complicate decision-making, and potentially lead to suboptimal actions due to misinformation, with the risk of triggering inappropriate parenting behaviors [5]. Furthermore, idealized images of parenting that proliferate on social media can create unrealistic expectations and pressures that undermine trust [10]. In addition, the “filter bubble” effect, where individuals are exposed only to information that aligns with their preferences and values, can promote biased parenting styles and isolate them from diverse viewpoints [11]. These challenges within the online information space pose the risk of complicating problem-solving processes in real-life parenting scenarios. Thus, online information related to parenting may have both positive and negative effects on problem-solving and the establishment of parenting styles. There is a need to accumulate knowledge that can help parents overcome the disadvantages of online information while maximizing its benefits in the context of parenting.

To examine the effective use of online information in parenting, it is critical to examine the interplay between online and face-to-face information. One explanatory model for the relationship between digital device use and human activity and well-being is the displacement-interference-complementarity (DIC) framework [12]. This framework offers a comprehensive understanding of the impact of digital devices on parenting by identifying 3 potential effects: displacement, interference, and complementarity. The displacement hypothesis suggests that digital devices may replace other activities, explaining that online social relationships serving as sources of parenting information may sometimes substitute for those in face-to-face settings. The interference hypothesis posits that digital devices may disrupt ongoing activities, indicating that parents’ use of online information could hinder face-to-face information gathering and support. Both hypotheses suggest that digital devices may limit face-to-face interactions in parenting, potentially negatively affecting positive parenting outcomes. In contrast, the complementarity hypothesis suggests that digital devices may provide access to information not available through face-to-face social relations, allowing parents to comprehensively use both online and offline sources of information to support their parenting efforts. By using the DIC framework, evidence can be provided to demonstrate the contexts in which online information benefits parents, contributing to a better understanding of how parenting support can be enhanced in the digital age.

Academic knowledge focused on technological advances is lagging [13], and research focusing on the interactivity between online and offline parenting information remains limited. For parents in the digital age, referring to or using online information has become commonplace. However, the affinity for digital technology varies among individuals, even within the so-called digital native generation, indicating diverse preferences [14]. In parenting, the level of online information use may vary based on factors such as the gender of the parent and the age of the child (in months) [15]. In the context of health information, information-seeking behaviors are significantly influenced by personal subjective norms [16]. This suggests that parents may exhibit a variety of patterns in their use of parenting information, ranging from those who integrate both online and offline sources to those who prefer online information or have traditional values and, therefore, use online resources sparingly. Exploring patterns of parenting information use, considering both online and offline sources, can provide nuanced evidence about the efficacy and challenges of digital information in parenting in light of the DIC framework.

To understand which component of the DIC framework online information falls under, it is important to consider parents’ social relationships as a potential influencing factor. Isolated individuals may use online communities, such as SNSs, to compensate for limited face-to-face interactions [17], suggesting that parents with limited social relationships might use online information as a substitute for face-to-face support. In addition, strong social support is correlated with higher health literacy [18], which in turn is associated with an increased rate of online health information searches [19]. This suggests that parents with extensive social networks are likely to be more adept at using both online and offline information, with online resources supplementing face-to-face interactions.

One outcome of effectively using parenting information to address parenting challenges is improved parenting self-efficacy, which is defined as parents’ beliefs about their ability to influence their children in ways that promote health and success [20]. High parenting self-efficacy is associated with positive outcomes in the parent-child relationship, child development, and parental mental health, underscoring its clinical importance [21]. Identifying the patterns of online or offline parenting information use associated with high parenting self-efficacy could provide insights into information use that enhances parenting performance.

Accordingly, this study aimed to explore the various patterns of combined online and offline parenting information use among parents in the digital age, categorize these patterns, and examine their characteristics by comparing the levels of parental social support and self-efficacy. This study seeks to answer 3 questions in particular:

1. What are the distinct patterns of the combined use of online and offline parenting information?
2. What are the demographic characteristics of the identified information usage patterns?
3. Which of these patterns of combined information use are associated with higher levels of parental social support and parenting self-efficacy?

## Methods

### Design

This study used a cross-sectional design, and a close-ended internet-based questionnaire survey was administered. To ensure the quality and transparency of the research, the Checklist for Reporting of Survey Studies (CROSS) developed by Sharma et al [22] and the CHERRIES (Checklist for Reporting Results of Internet E-Surveys) developed by Eysenbach [23] were used. This paper reports a portion of the data from a survey titled “Information Behavior on Social Networking Services Among Parents of Infants and Toddlers.”

### Participants and Sampling

Convenience sampling was adopted for participant recruitment and was performed in collaboration with Cross Marketing Inc, an internet-based survey company that oversees nationwide sampling in Japan. With over 5 million active online users, Cross Marketing Inc provides academic survey services for diverse demographics. The panel managed by the company consists of a broad range of internet users, providing a large control group that is unbiased by specific sites or advertisements. In this study, 434 parents of children aged 0-3 years were recruited from the Cross Marketing Inc panel. As part of the recruitment process, a one-time survey invitation was sent by the research company to approximately 2000 randomly selected individuals deemed eligible for the study. Recipients who answered a preliminary question about their children’s age that was inconsistent with the study criteria were excluded. Those who agreed to participate in the study clicked “agree” after reading the research request document, which directed them to the internet-based survey link. The target sample size was set at over 200 participants each for fathers and mothers. Recruitment concluded once this target was reached. However, some individuals submitted late responses after recruitment ended, resulting in a final number of respondents that exceeded the target.

### Data Collection

In August 2023, data were collected through an internet-based survey involving those directed through the aforementioned process. The survey was created and administered by the authors using the Qualtrics online survey system, which ensures data security through mechanisms such as encryption, redundancy, continuous monitoring, and single sign-on, and is FedRAMP certified and ISO27001 accredited. The survey was user-friendly, designed by the authors, and pretested to avoid design problems. Participants who completed the survey received reward points or other rewards according to the protocols of Cross Marketing Inc.

### Measures

The survey items used in the internet-based survey are presented in Multimedia Appendix 1.

#### Frequency of Use of Parenting Information Resources

In this study, the selection of parenting information resources used by parents in today’s digital society was based on the concept of the information space surrounding parents of young children by Xie et al [3]. Parents’ information space consists of offline and online communities defined by proximity and the broader public sphere—the lifeworld. Online communities include everyday online spaces such as web search platforms, SNSs, and online parenting communities. Offline communities represent more immediate everyday spaces that include family members, friends, and parenting peers. The lifeworld includes broader public institutions and services, both online and offline, including medical institutions and their websites. Accordingly, in this study, the categorization followed was “online resources” from online communities and lifeworlds and “offline resources” from offline equivalents. Online sources of parenting information were divided into websites, SNSs, and parenting-related applications. Websites included child- and parent-related websites, blogs, and hospital homepages. Given the Japanese context, SNSs included platforms such as X (formerly known as Twitter), Instagram, Facebook (Meta Platforms), and YouTube (Google), excluding LINE (LY Corporation) because of its primary use for personal communication and limited feed functionality. Parenting apps included those useful for pregnancy tracking, baby development updates, and immunization schedules. Offline resources were identified as family, friends, parenting peers, and professionals (including health and education professionals), who provided information from offline communities and lifeworlds. Participants rated their subjective frequency of use of 6 information sources on a 6-point scale from “1: Never use” to “6: Always use.”

#### Parenting Self-Efficacy

The Parenting Self-efficacy Scale [24], which was developed and validated in the Japanese context, was used. It consists of 13 items rated on a 5-point Likert scale, with higher scores indicating greater parenting self-efficacy. The Cronbach alpha for this study was 0.85.

#### Parental Social Support

Drawing on the classification of social support proposed by previous research [25], I developed items based on another previous study in the context of parenting [26], focusing on informational and emotional support. It is assumed that the availability of support varies depending on the source; therefore, I established 4 categories of respondents: spouses (including partners), other family members, friends and parenting peers, and professionals. A total of 8 items were created to reflect both types of support. Respondents evaluated these items using a 4-point scale ranging from “1: Strongly disagree” to “4: Strongly agree.” In cases where the designated respondent category did not apply (eg, if the participant was a single parent), respondents were instructed to select “0: Not applicable.”

#### Participant Demographics

Data were collected on participants’ age, number of children, age of the youngest child, occupational status, educational level, and subjective economic status.

### Statistical Analysis

First, descriptive statistics for both fathers and mothers were conducted to examine differences in the frequency of information source usage and social support related to parenting. Next, the partitioning around medoids (PAM), a nonhierarchical clustering method applicable to ordinal scales, was used to identify patterns in gathering parental information. This approach addresses traditional challenges of hierarchical clustering, which may hinder finding optimal solutions because participants remain in specific clusters throughout all analysis steps. Unlike the k-means method, which relies on means, PAM uses actual data points (medoids) as cluster centers, maintaining rank information and providing robust clustering results in the presence of outliers or noise. Initial medoids are determined using a Greedy algorithm, which sequentially selects locally optimal solutions to minimize the total cost (the sum of distances from each data point to the nearest medoid), effectively reflecting the data’s structure. The Manhattan distance was adopted as the distance metric, which, unlike Euclidean distance, represents movement along each dimension rather than straight-line distance, offering robustness against outliers and noise.

The number of clusters was determined based on theoretical and practical interpretability, along with results from the elbow method and gap statistic, both indicators of clustering appropriateness. The elbow method visualizes how the sum of squared errors (SSE) within clusters decreases as the number of clusters (k) increases, with the “elbow” point indicating the optimal number of clusters. In this study, SSE was plotted for values of k from 1 to 20 to locate the elbow. The gap statistic method compares the clustering results of observed data with those of randomly generated data to determine the optimal number of clusters, identifying the maximum gap statistic as optimal and indicating how much better the clustering results are compared with random data. A maximum of 500 bootstrap iterations was conducted for the observed data set from 1 to 20 clusters.

Initially, we conducted separate analyses for the father and mother, confirming similarities in their clustering. Given the observed similarities between both parents, the measures taken to reduce the sample size for each cluster, and the importance of integrating the clusters, we decided to consolidate all samples for cluster analysis. The Kruskal-Wallis test and the Steel-Dwass test were used for multiple comparisons to identify clusters based on the reference variables.

To investigate the association between the extracted clusters and demographic characteristics, as well as the availability of parental social support, we conducted the analysis using the Kruskal-Wallis test, Steel-Dwass test, or chi-square test. In addition, to compare parenting self-efficacy across clusters, 1-way ANOVA and Tukey honest significant difference (HSD) tests were performed for multiple comparisons. Statistical analyses were conducted using JMP Pro (version 17.0; SAS Institute) and R language (R Foundation for Statistical Computing), with a significance level set at 5%.

The required sample size for 1-way ANOVA was calculated using G-power, considering an effect size (f) of 0.25 (medium), α error probability of 0.05, power of 0.8, and 20 groups, resulting in a minimum required sample size of 360. The number of groups was based on the maximum expected in PAM for this study. Missing values in parenting self-efficacy were imputed using least squares prediction based on nonmissing values for each scale when less than half of the data were missing.

### Ethical Considerations

Participants were provided written information about the following aspects related to the study and their participation: an overview of the study, voluntary participation, no penalty for nonparticipation, their right to refuse to answer, maintenance of anonymity, use of data only for research purposes, and strict management of personal information. Consent to participate was obtained when participants selected the consent button in the internet-based survey. All questions included the option for participants to refuse to answer if they preferred. This study was conducted following approval received from the Institutional Ethics Committee, the ethics committee for research involving human participants at Toyama Prefectural University (August 10, 2023, approval number: R5-16).

## Results

### Descriptive Statistics

Of the respondents who returned the questionnaire, data from 420 individuals—those without missing data on items related to the use of information sources in parenting—were included in the analysis. The basic demographic characteristics of the participants are presented in Table 1. The sample consisted of 201/420 mothers (47.9%) and 219/420 fathers (52.1%). The distribution of the Parenting Self-Efficacy Scale exhibited a skewness of –0.35 and a kurtosis of 0.14. None of the scales violated the thresholds (skewness: absolute value >2; kurtosis: absolute value >7) [27], which could potentially cause bias in parametric analyses.

**Table 1:** Demographics.

				Mothers (N=201)				Fathers (N=219)		
				n (%)		Mean (SD)		n (%)		Mean (SD)
**Demographics**										
	Parent’s age (n=388)		—^a^		34.5 (4.89)		—		39.8 (6.57)	
	**Number of children**									
		One	93 (0.46)		—		90 (0.41)		—	
		Two	83 (0.41)		—		91 (0.42)		—	
		Three or more	25 (0.12)		—		38 (0.17)		—	
	**Youngest child’s age**									
		Infant	69 (0.34)		—		52 (0.24)		—	
		1 year old	62 (0.31)		—		73 (0.33)		—	
		2 years old	41 (0.2)		—		56 (0.26)		—	
		3 years old	29 (0.14)		—		38 (0.17)		—	
	**Cohabiting family members**									
		Partner	192 (0.96)		—		214 (0.98)		—	
		Own family members	12 (0.06)		—		13 (0.94)		—	
		Partner's family members	9 (0.05)		—		4 (0.02)		—	
	**Occupational status**									
		Full-time worker	58 (0.29)		—		215 (0.98)		—	
		Part-time worker	23 (0.11)		—		1 (0)		—	
		Homemaker	73 (0.36)		—		1 (0)		—	
		On maternity or childcare leave	47 (0.23)		—		2 (0.01)		—	
	**Educational level (n=418)**									
		Junior high school or High school graduate	24 (0.12)		—		27 (0.12)		—	
		Junior college or Vocational school graduate	61 (0.31)		—		21 (0.1)		—	
		University or Graduate school graduate	115 (0.58)		—		170 (0.78)		—	
	**Subjective economic status**									
		Very concerned	22 (0.11)		—		32 (0.15)		—	
		Somewhat concerned	83 (0.41)		—		86 (0.39)		—	
		Slightly concerned	82 (0.41)		—		93 (0.42)		—	
		Not concerned at all	14 (0.07)		—		8 (0.04)		—	
	**Having digital devices**									
		Having smartphones	200 (1)		—		215 (0.98)		—	
		Having tablets	45 (0.22)		—		70 (0.32)		—	
		Having computers	85 (0.42)		—		146 (0.67)		—	
Parenting efficacy (n=418)				—		3.48 (0.67)		—		3.50 (0.66)

^a^Not applicable.

### Gender Differences in Parental Information Use and Availability of Support

The frequency of using information sources in parenting and descriptive statistics for parental social support are presented in Multimedia Appendix 2. Among the parenting-related information sources, mothers more frequently used websites (*P*=.003), SNSs (*P*<.001), parenting apps (*P*<.001), and friends or parenting peers (*P*<.001) compared with fathers. Regarding parental social support, fathers perceived greater availability of informational support (*P*<.001) and emotional support (*P*=.02) from their partners compared with mothers. Conversely, mothers perceived greater availability of emotional support from friends than fathers (*P*=.01).

### Cluster Analysis

**Figure 1:**
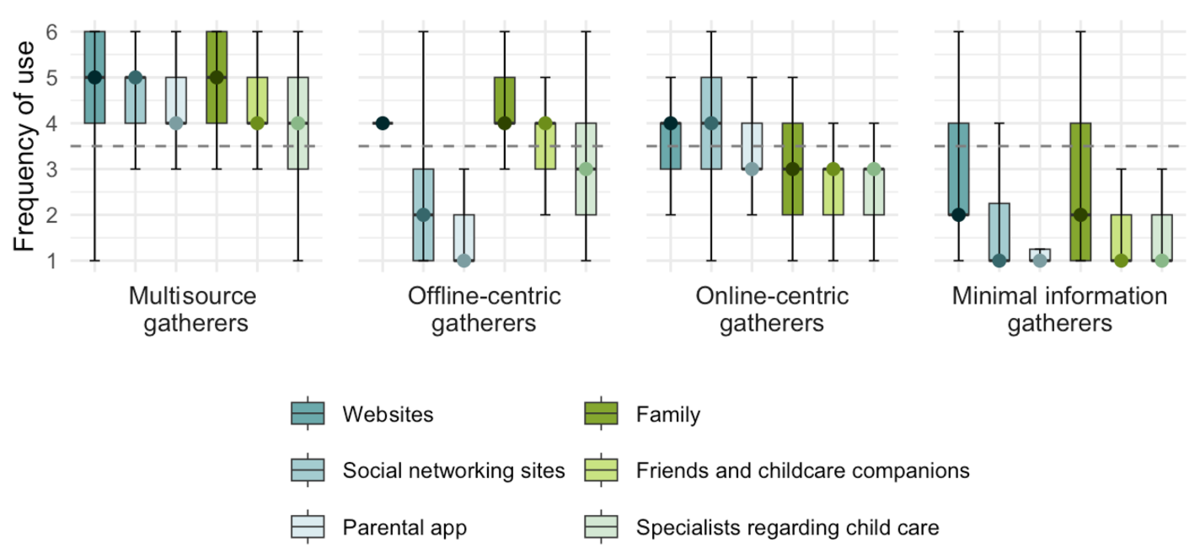
Clusters of use patterns of parenting information resources.

Based on the clusters extracted by PAM, the elbow method was applied. Although a clear elbow point could not be definitively established, the reduction in the total within-cluster sum of squares became more gradual at k=4 (Multimedia Appendix 3). The gap statistic plot indicated that k=4 yielded the maximum value, followed by a slight decrease (Multimedia Appendix 4). Consequently, we decided to proceed with a cluster count of 4 (Figure 1 and Multimedia Appendix 5). The clusters were named based on the Kruskal-Wallis test and the Steel-Dwass test, as follows: Cluster 1, with a high frequency of use of all information sources, was named “Multisource gatherers (n=161).” Cluster 2, with a relatively higher frequency of use of offline sources compared with online sources, was named “Offline-centric gatherers (n=105).” Cluster 3, with a relatively higher frequency of use of online sources compared with offline sources, was named “Online-centric gatherers (n=86).” Cluster 4, with an extremely low frequency of use of all information sources, was named the “Minimal information gatherers (n=68).”

### Differences of Demographics by Parent Information Use Patterns

The comparison results of the demographics of the 4 clusters are presented in Table 2. “Multisource gatherers” are relatively younger, have more mothers, fewer children aged 2, more often cohabitate with spouses, exhibit a higher prevalence of maternity and parental leave, and enjoy a better living situation. “Offline-centric gatherers” are relatively older, have more fathers, tend to have more families with two children rather than one, and predominantly work full-time with less maternity and parental leave; they also have a higher rate of university graduates. “Online-centric gatherers” tend to have more infants as their youngest children and fewer 3-year-olds, work more part-time, and have a higher proportion of junior college or vocational graduates, with fewer holding university degrees. “Minimal information gatherers” are relatively characterized by more fathers, a higher number of 2-year-olds, and a predominance of full-time employment.

**Table 2:** Demographic differences by parental information-use patterns.

			1. Multisource gatherers (n=161)					2. Offline-centric gatherers (n=105)					3. Online-centric gatherers (n=86)					4. Minimal information gatherers (n=68)					*F*^a^ test (*df*)				Chi-square (*df*)^b^				*P* value				Multiple comparisons^c^	
			n (%)		Mean (SD)		n (%)			Mean (SD)		n (%)			Mean (SD)		n (%)			Mean (SD)																
Parent’s age (n=388)			—^d^		35.51 (5.81)		—			39.7 (5.88)		—			36.2 (7.14)		—			38.7 (5.89)		11.20 (3,384)				—				<.001				2≧4≧3≧1		
**Gender**																								—				31.09 (3)				<.001				—
	Mother	95 (0.59)^e^		—		36 (0.34)			—		51 (0.59)			—		19 (0.28)			—																	
	Father	66 (0.41)		—		69 (0.66)^e^			—		35 (0.41)			—		49 (0.72)^e^			—																	
**Number of children**																								—				16.36 (6)				.01				—
	One	74 (0.46)		—		29 (0.28)^f^			—		45 (0.52)			—		35 (0.51)			—																	
	Two	62 (0.39)		—		55 (0.52)^e^			—		32 (0.37)			—		25 (0.37)			—																	
	Three or more	25 (0.16)		—		21 (0.2)			—		9 (0.10)			—		8 (0.12)			—																	
**Youngest child’s age**																								—				42.31 (9)				<.001				—
	Infant	54 (0.34)		—		16 (0.15)			—		36 (0.42)^e^			—		15 (0.22)			—																	
	1 year old	58 (0.36)		—		39 (0.37)			—		22 (0.26)			—		16 (0.24)			—																	
	2 years old	20 (0.12)^f^		—		28 (0.27)			—		21 (0.24)			—		28 (0.41)^e^			—																	
	3 years old	29 (0.18)		—		22 (0.21)			—		7 (0.08)^f^			—		9 (0.13)			—																	
**Cohabitating family members**																								—				8.13 (3)				.04				—
	Partner	159 (0.99)^e^		—		101 (0.96)			—		80 (0.93)			—		66 (0.97)			—																	
	Own family members	16 (0.10)		—		4 (0.04)			—		4 (0.05)			—		1 (0.01)			—																	
	Partner's family members	9 (0.06)		—		1 (0.01)			—		2 (0.02)			—		1 (0.01)			—																	
**Occupational status**																								—				34.66 (9)				<.001				—
	Full-time worker	88 (0.55)^f^		—		80 (0.76)^e^			—		53 (0.62)			—		52 (0.76)^e^			—																	
	Part-time worker	8 (0.05)		—		4 (0.04)			—		10 (0.12)^e^			—		2 (0.03)			—																	
	Homemaker	34 (0.21)		—		19 (0.18)			—		11 (0.13)			—		10 (0.15)			—																	
	On maternity or childcare leave	31 (0.19)^e^		—		2 (0.02)^f^			—		12 (0.14)			—		4 (0.06)			—																	
**Educational level (n=418)**																								—				14.92 (6)				.02				—
	Junior high school or High school graduate	21 (0.13)		—		7 (0.07)			—		11 (0.13)			—		12 (0.18)			—																	
	Junior college or Vocational school graduate	33 (0.20)		—		14 (0.13)			—		25 (0.29)^e^			—		10 (0.15)			—																	
	University or Graduate school graduate	107 (0.66)		—		83 (0.80)^e^			—		49 (0.58)^f^			—		46 (0.68)			—																	
**Subjective economic status^g^**																								—				10.10 (3)				.02				1≧2=3=4
	Very concerned	12 (0.07)		—		12 (0.11)			—		13 (0.15)			—		17 (0.25)			—																	
	Somewhat concerned	68 (0.42)		—		38 (0.36)			—		36 (0.42)			—		27 (0.40)			—																	
	Slightly concerned	69 (0.43)		—		52 (0.5)			—		33 (0.38)			—		21 (0.31)			—																	
	Not concerned at all	12 (0.07)		—		3 (0.03)			—		4 (0.05)			—		3 (0.04)			—																	

^a^Results of 1-way ANOVA.

^b^Results of chi-square test or Kruskal-Wallis test.

^c^The result of Steel-Dwass test or Turkey HSD test.

^d^Not applicable.

^e^Indicates significant differences observed in the residual analysis and denotes a significantly higher proportion. +

^f^Indicates significant differences observed in the residual analysis and denotes a significantly lower proportion. -

^g^The larger the value, the less concern there is about the economic situation.

### Comparison of Parental Social Support Based on Parenting Information Use Patterns

**Figure 2:**
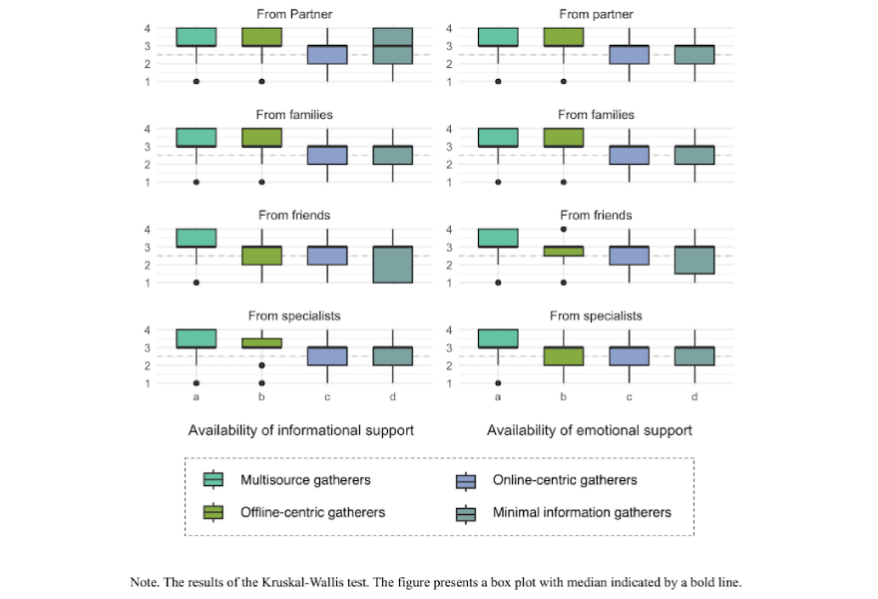
Comparison of parental social support based on parenting information use patterns.

The comparative results of the availability of social support in childcare across the 4 clusters are presented in Figure 2 and Multimedia Appendices 6 and 7. A significant relationship was confirmed between all types of available support and clusters. Regarding spousal support, both informational and emotional aspects were more available to “Multisource gatherers” compared with “Online-centric gatherers.” For family support, both informational and emotional aspects were more available to “Multisource gatherers” and “Offline-centric gatherers” compared with “Online-centric gatherers” and “Minimal information gatherers.” In terms of support from friends and parenting peers, both informational and emotional aspects were higher for “Multisource gatherers” compared with “Online-centric gatherers” and “Minimal information gatherers,” while “Offline-centric gatherers” also had more availability than “Minimal information gatherers.” Finally, support from professionals showed greater availability in both informational and emotional aspects for “Multisource gatherers” compared with “Online-centric gatherers” and “Minimal information gatherers.”

### Comparison of Parenting Self-Efficacy Based on Parenting Information Use Patterns

**Figure 3:**
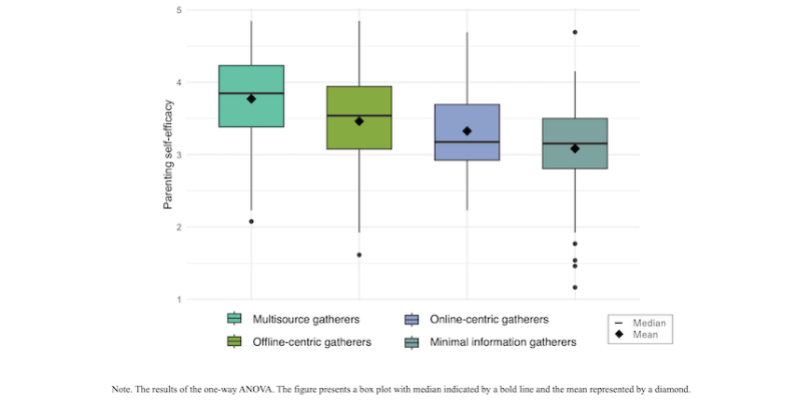
Comparison of parenting self-efficacy based on parenting information use patterns.

The results comparing parenting self-efficacy based on parenting information usage patterns are presented in Figure 3 and Multimedia Appendix 8. The 1-way ANOVA revealed a significant relationship between parenting information usage patterns and parenting self-efficacy (*F*_3,414_=22.86, *P*<.001). According to the Tukey HSD test, “Multisource gatherers” demonstrated significantly higher parenting self-efficacy compared with “Offline-centric gatherers,” “Online-centric gatherers,” and “Minimal information gatherers” (*P*<.001 for each). In addition, “Offline-centric gatherers” exhibited significantly higher self-efficacy than “Minimal information gatherers” (*P*<.001).

## Discussion

### Principal Findings

This study identified 4 types of parenting information use patterns among parents of infants and toddlers and elucidated their characteristics through comparisons of parental social support and parenting self-efficacy. The main findings of the study are discussed below.

### Multisource Type

First, the “Multisource gatherers” tended to use both online and face-to-face information sources frequently. This suggests that online information resources may function as a complement for these groups, according to the DIC framework [12]. Parents of this type had higher levels of support from family, friends, and specialists related to parenting than did “Online-centric gatherers” and “Minimal information gatherers.” This suggests that they may have extensive social networks that allow them to access a wide range of information sources for parenting. In addition, individuals in this type had higher levels of parenting self-efficacy than those in the other types. Access to diverse information can contribute to problem-solving when information utilization skills are high; otherwise, it can lead to confusion owing to information overload [4]. These parents may effectively use diverse information from both offline and online sources to solve parenting challenges because of their information use and social relationship-building skills, which may contribute to higher parenting self-efficacy. However, given the cross-sectional design of this study, reverse causality is also possible. Parents with high parenting self-efficacy may experience a greater sense of reassurance when using smart devices to alleviate parenting anxiety, leading to reduced anxiety [28]. High parenting self-efficacy, which is associated with reduced psychological distress in parenting [29], may mitigate the negative aspects of online information, such as psychological distress from social comparisons on SNSs [30], enabling parents to use multichannel information effectively.

This type has a relatively high proportion of mothers and individuals on maternity or parental leave and a low proportion of full-time workers. In addition, this type, along with “Online-centric gatherers,” exhibits a high frequency of online information usage, which aligns with previous research indicating that women engage more frequently in internet use and the exchange of social support through SNSs [31,32]. Furthermore, working mothers are expected to have a broader social network due to their workplace connections compared with stay-at-home mothers. Mothers on maternity or parental leave are more likely to have more time to use their parenting networks than those currently used. As a result, it can be inferred that mothers on maternity or parental leave were leveraging both online and offline information to address parenting challenges.

### Online Type

“Online-centric gatherers,” who exhibit a relatively higher frequency of use of online information sources centered around SNSs compared with offline sources, tend to show lower availability of informational and emotional social support in parenting compared to “Multisource gatherers” and “Offline-centric gatherers.” This type may be using online information to replace limited face-to-face social relationships, suggesting that online information acts as a substitute, according to the DIC framework [12]. The lower parenting self-efficacy observed in this type, compared to “Multisource gatherers,” suggests that substituting face-to-face information with online information may be limited to offer positive outcomes in parenting. Previous research has suggested that using digital resources as a substitute for family support in solving parenting challenges may lead to low parental role achievement [26]. The results of this study are consistent with previous research and strengthen the evidence regarding the effectiveness of online information as a complement to and its limitations as a substitute for offline information in resolving parenting challenges. Individuals struggling with social isolation or loneliness may have a high affinity for online information from SNSs [17], suggesting that parents of this type may have limited face-to-face social relations and may intentionally or unintentionally rely on online information to cope with these issues.

This type is characterized by a relatively high proportion of mothers with infants, part-time workers, and individuals with associate degrees. During the infant stage, the emphasis on caregiving is greater compared with the toddler stage, resulting in more restrictions in daily parenting life. This may have led to the use of online parenting networks, which are more easily accessible than in-person support. In addition, previous research [28] indicates that highly educated mothers tend to experience anxiety related to problem-solving in parenting when using smart devices. Parents of this type, owing to their higher educational status, may recognize concerns regarding online information, making them less likely to adopt an information use style that is heavily biased toward online sources.

### Offline Type

“Offline-centric gatherers” preferred face-to-face to online sources of information. This type, likely a conventional type from the pre-SNS era, appeared to prioritize information obtained through face-to-face interactions for parenting. Despite the proliferation of digital technology and the tech-savvy population, there is a certain proportion of nonadopters who either intentionally or unintentionally choose not to use technology [33,34]. As such, those in the offline group may be similar to nonadopters of digital technology. Compared with the “Online-centric gatherers” and “Minimal information gatherers,” this type perceived greater availability of support from family and friends and appeared to rely on ample face-to-face resources for parenting without the need for online information. However, this type exhibited lower parenting self-efficacy compared with “Multisource gatherers.” This suggests that online information, as a complementary hypothesis, may contribute to higher levels of parenting self-efficacy. Using online information in this context may help further enhance the parenting self-efficacy of these parents.

This type is characterized by a relatively high proportion of fathers, parents with only one child, full-time workers, and individuals with university or graduate degrees. Compared with pregnant women and mothers, there are limited SNSs and peer support opportunities for fathers related to parenting [35], which may restrict their ability to expand online parenting networks and effectively use online information. In addition, the prevalence of highly educated individuals in this type is consistent with the aforementioned tendency for online use in problem-solving to evoke negative emotions [28].

### Minimal Information Gatherers

“Minimal information gatherers” exhibited a generally low frequency of information use. This type recognized the availability of support from family, friends, and professionals to a lesser extent, and their self-efficacy in parenting was lower than that of all other types. This type may have limited access to all parenting information resources and an extremely narrow social network, both within and outside the family, indicating potential problems in social relations. Social isolation is a significant factor in worsening parental mental health [36] and a risk factor for maltreatment [37]. This type may represent a high-risk case that requires assistance from experts.

In this group, there was a high proportion of fathers and full-time workers among the samples. In Japanese society, owing to the influence of gender role division, mothers often take on the central role in child-rearing, while fathers’ involvement may be limited in some households [38]. Busy fathers, who may have less active involvement, might lack sufficient parenting experience, which could lead to lower self-efficacy in child-rearing. In addition, this group uniquely recognized the availability of informational support from partners as comparable with “Multisource gatherers” and “Offline-centric gatherers” while acknowledging the lower availability of emotional support from partners compared with the other two types. This implies that, despite problems in the emotional relationship between couples, the limited social networks they possess confine their sources of parenting information to their partners. Fathers may struggle more than mothers to distinguish negative emotions stemming from marital discord from those associated with parenting, which supports the “Fathering Vulnerability Hypothesis [39].” Considering this hypothesis, fathers in this category might experience complex negative emotions due to their reliance on partners for parenting information despite having emotional challenges in their relationships, thereby reinforcing negative experiences in parenting and leading to a decline in self-efficacy.

### Implications

The results confirm that the DIC framework is generally applicable in the context of parenting information use. As those with the highest levels of parenting self-efficacy are “multisource gatherers,” it is recommended that parents use online information as a complement to face-to-face advice when seeking parenting information. On the other hand, it should not be used as a substitute for face-to-face information provision, as it is less likely to lead to favorable outcomes. However, parents who rely on online information may face potential issues, such as limited social networks. Therefore, simply reducing the use of online information does not guarantee an improvement in parenting outcomes.

Parenting support professionals are required to provide customized assistance based on the types of parenting information use identified in this study. “Multisource gatherers” are encouraged to enhance their ability to use diverse information sources, both online and offline. For “Online-centric gatherers,” there is a need to strengthen information literacy to assess the reliability and quality of online information, particularly given the variability in these factors in the short term. For example, reports indicate that interactive websites, parent-created sites, and sites originating from South America have significant room for improvement in ethical and content aspects [40]; therefore, parents should be cautious when using them. In the medium to long term, promoting enriched social relationships in parenting to facilitate a transition to “Multisource gatherers” would be effective. For “Offline-centric gatherers,” having robust offline social networks is crucial, and supporting the strengthening of these networks to obtain valuable informational support from family and community is beneficial. For “Minimal information gatherers,” identifying underlying issues, such as family discord or social isolation, and providing tailored professional support is effective. By addressing these underlying issues and enhancing social relationships in parenting may broaden information use patterns in parenting, leading to a transition to other categories.

### Limitations

A few limitations of this study must be noted. First, causal inferences could not be made, given its cross-sectional design. Second, owing to the self-report questionnaire format, the possibility of information bias cannot be dismissed. Third, the convenience sampling approach adopted, collaborating with a specific survey company in Japan, does not eliminate the possibility of selection bias. In particular, online survey respondents may be biased toward higher online affinity and information literacy, thus limiting the generalizability of the results. Fourth, it is difficult to completely rule out the possibility of different clusters emerging from this study. It is crucial to confirm the reproducibility of the clusters identified in this study through cluster analysis using a larger, randomly sampled data set with more rigorous procedures. Fifth, this study did not consider information literacy when capturing the characteristics of the types of parenting information use patterns. Finally, the measure examining the availability of parenting support to investigate the characteristics of the clusters is a single-item scale, and its reliability and validity are not sufficiently guaranteed. It is also important to note the existence of parenting support networks, such as workplace colleagues, which could not be measured in this study.

Future research involving larger international samples and different methodologies not influenced by online affinity may increase the robustness of this study’s findings. In addition, it is expected that patterns of information use may change under the influence of various factors such as parents’ educational experiences and children’s age. Identifying the process of change and the factors involved could contribute to a more nuanced understanding of the characteristics of parenting information used in today’s digital society.

### Conclusion

This study identified types of parenting information-seeking patterns among parents in today’s digital society and explored their characteristics through comparisons of parental social support and parenting self-efficacy. The results indicated the following information-seeking patterns among parents: those who use both online and face-to-face information sources in a multichannel, hybrid manner; those with a bias toward either online or face-to-face information; and those with a generally low frequency of parenting information use. The type that used both online and offline resources exhibited the highest levels of parenting self-efficacy. The results of this study support the DIC framework and strengthen the evidence for the effectiveness of online information as a complement to and its limitations as a substitute for offline information in resolving parenting challenges. Parenting support professionals are encouraged to understand parents’ current information use strategies and actively foster their social relationships, helping them to adopt more diverse and comprehensive approaches to information use.

## Appendix

Multimedia Appendix 1 Survey items.

Multimedia Appendix 2 Gender differences in information and social support availability in child care.

Multimedia Appendix 3 The elbow methods.

Multimedia Appendix 4 The gap statistic method.

Multimedia Appendix 5 Cluster analysis results.

Multimedia Appendix 6 Comparison of availability of support by parenting information use patterns.

Multimedia Appendix 7 Results of multiple comparisons using Steel-Dwass test in the comparison of availability of supports across parenting information use patterns.

Multimedia Appendix 8 Results of multiple comparisons using Tukey's honest significant difference test in the comparison of parenting self-efficacy across parenting information use patterns.

## Abbreviations

CHERRIES: Checklist for Reporting Results of Internet E-Surveys
CROSS: Checklist for Reporting of Survey Studies
DIC: displacement–interference–complementarity
HSD: honest significant difference
PAM: partitioning around medoids
SNS: social networking site
SSE: sum of squared errors
