# Supplementary material for: Parental Information-Use Strategies in a Digital Parenting Environment and Their Associations With Parental Social Support and Self-Efficacy: Cross-Sectional Study
Source: JMIR Pediatr Parent. 2024 Dec 19;7:e58757. doi: 10.2196/58757 (PMC11695971; doi:10.2196/58757)
Supplement: Multimedia Appendix 1 [file pediatrics_v7i1e58757_app1.pdf]

Multimedia Appendix 1.

The survey items included in the online questionnaire administered in this study are presented below. Each question included the answer option "do not wish to answer."

Question: Your age

( ) years old

Question: Your sex

1. Male 2. Female 3. Other

Question: Number of children

( ) children

Question: Age of your youngest child

( ) Years ( ) Months

Question: Please select all other family members living with you besides your children

1. Spouse (including partner)
2. Your parents
3. Spouse's (including partner's) parents
4. Your siblings
5. Spouse's (including partner's) siblings
6. Others ( )

Question: Your employment status

1. Full-time 2. Part-time 3. Homemaker 4. On maternity/paternity leave

Question: Highest level of education

1. Junior high school graduate 2. High school graduate
3. Vocational school graduate 4. Junior college graduate
5. University graduate 6. Graduate school graduate 6. Other ( )

Question: Living situation

1. Very worried 2. Somewhat worried 3. Not so worried 4. Not worried at all

We will now ask about the sources of information you use for parenting.

Question: How often do you use the following sources to obtain parenting-related information?

	Never use	Hardly use	Rarely use	Sometimes use	Often use	Always use
Sources of face-to-face information						
Family (spouse, parents, siblings, etc.)	1	2	3	4	5	6
Friends/Parenting peers	1	2	3	4	5	6
Professionals (doctors, public health nurses, midwives, childcare workers, kindergarten teachers, etc.)	1	2	3	4	5	6
Sources of online information						
Websites (hospital homepages, sites related to children and parenting, blogs, etc.)	1	2	3	4	5	6
SNSs (Twitter, Instagram, TikTok, YouTube, Facebook, LinkedIn, etc.) *excluding LINE	1	2	3	4	5	6
Parenting-related apps (pregnancy week trackers, baby development, vaccination schedules, etc.)	1	2	3	4	5	6

Question: Please describe the support you receive regarding parenting.

Item	Strongly disagree	Somewhat disagree	Somewhat agree	Strongly agree	Not applicable
I can obtain advice about parenting from my spouse (including partner).	1	2	3	4	0
I can obtain advice about parenting from family members other than my spouse (including partner).	1	2	3	4	0
I can obtain advice about parenting from friends or other mothers.	1	2	3	4	0
I can obtain advice about parenting from professionals (such as doctors, public health nurses, childcare workers, etc.).	1	2	3	4	0
I can receive emotional understanding, empathy, and a sense of security from my spouse (including partner) regarding parenting.	1	2	3	4	0
I can receive emotional understanding, empathy, and a sense of security from family members other than my spouse (including partner) regarding parenting.	1	2	3	4	0
I can receive emotional understanding, empathy, and a sense of security from friends or other mothers regarding parenting.	1	2	3	4	0
I can receive emotional understanding, empathy, and a sense of security from professionals (such as doctors, public health nurses, childcare workers, etc.) regarding parenting.	1	2	3	4	0

Question: Please select one number for the following statements that applies to you.

	Item	Do not think so	Do not think much	Neutral	Somewhat think so	Think so
	I think I can handle any problems I may have in raising my children.	1	2	3	4	5
	I can control my emotions.	1	2	3	4	5
	I can express my feelings straightforwardly.	1	2	3	4	5
	I can share the joy of parenting with those close to me.	1	2	3	4	5
	People around me approve of my parenting.	1	2	3	4	5
	If I need help raising my child, I can rely on others to help me.	1	2	3	4	5
	I can seek advice from others on parenting.	1	2	3	4	5
	I think there are things that only I can do to help raise my child.	1	2	3	4	5
	I feel free to talk to people.	1	2	3	4	5
	I am able to make friends while raising children.	1	2	3	4	5
	I have time to do things other than raise my children.	1	2	3	4	5
	I lack confidence in continuing to raise children.	1	2	3	4	5
	I have my own image of childcare.	1	2	3	4	5
