# Supplementary figures and images for: Parental Information-Use Strategies in a Digital Parenting Environment and Their Associations With Parental Social Support and Self-Efficacy: Cross-Sectional Study
Source: JMIR Pediatr Parent. 2024 Dec 19;7:e58757. doi: 10.2196/58757 (PMC11695971; doi:10.2196/58757)

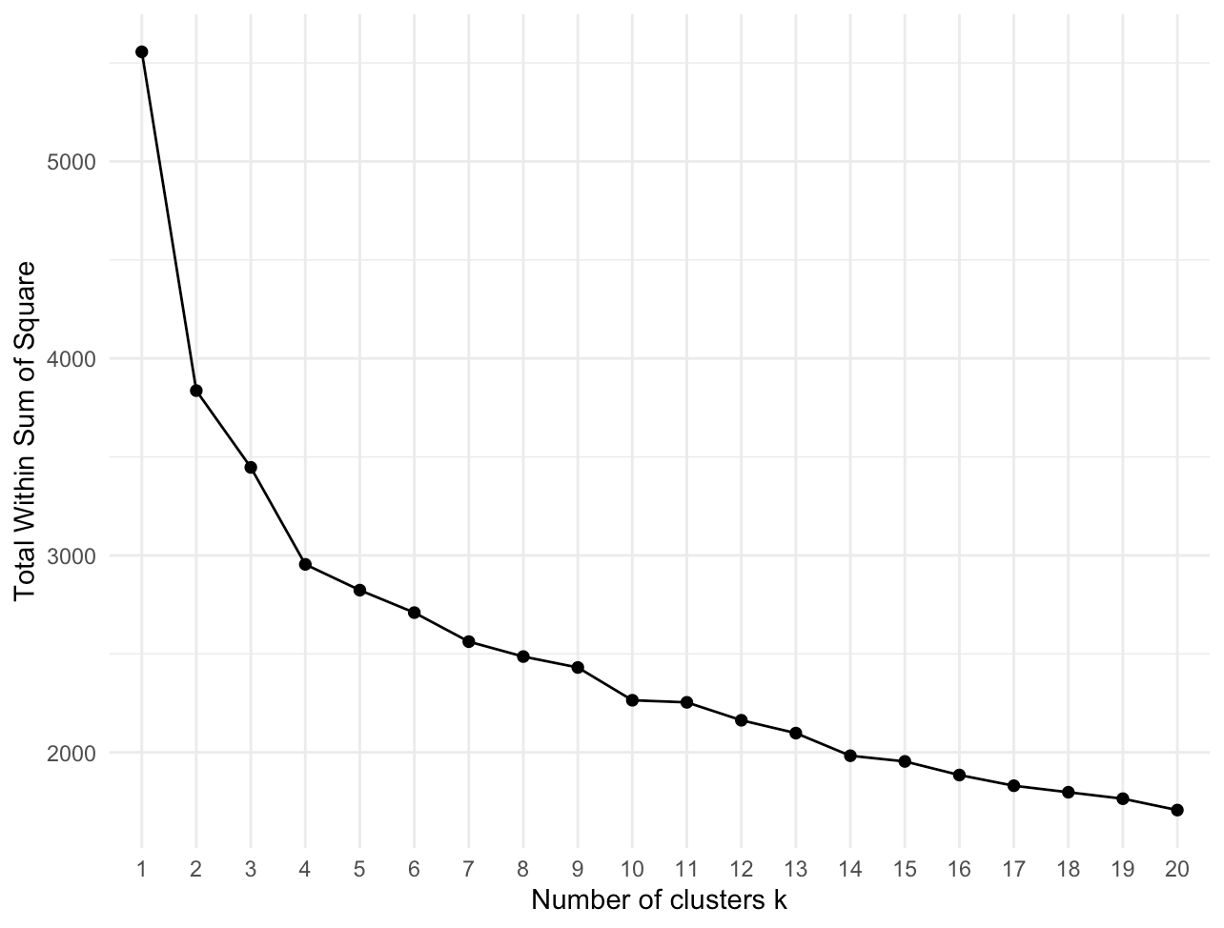

Supplement: Multimedia Appendix 3 [file pediatrics_v7i1e58757_app3.png]

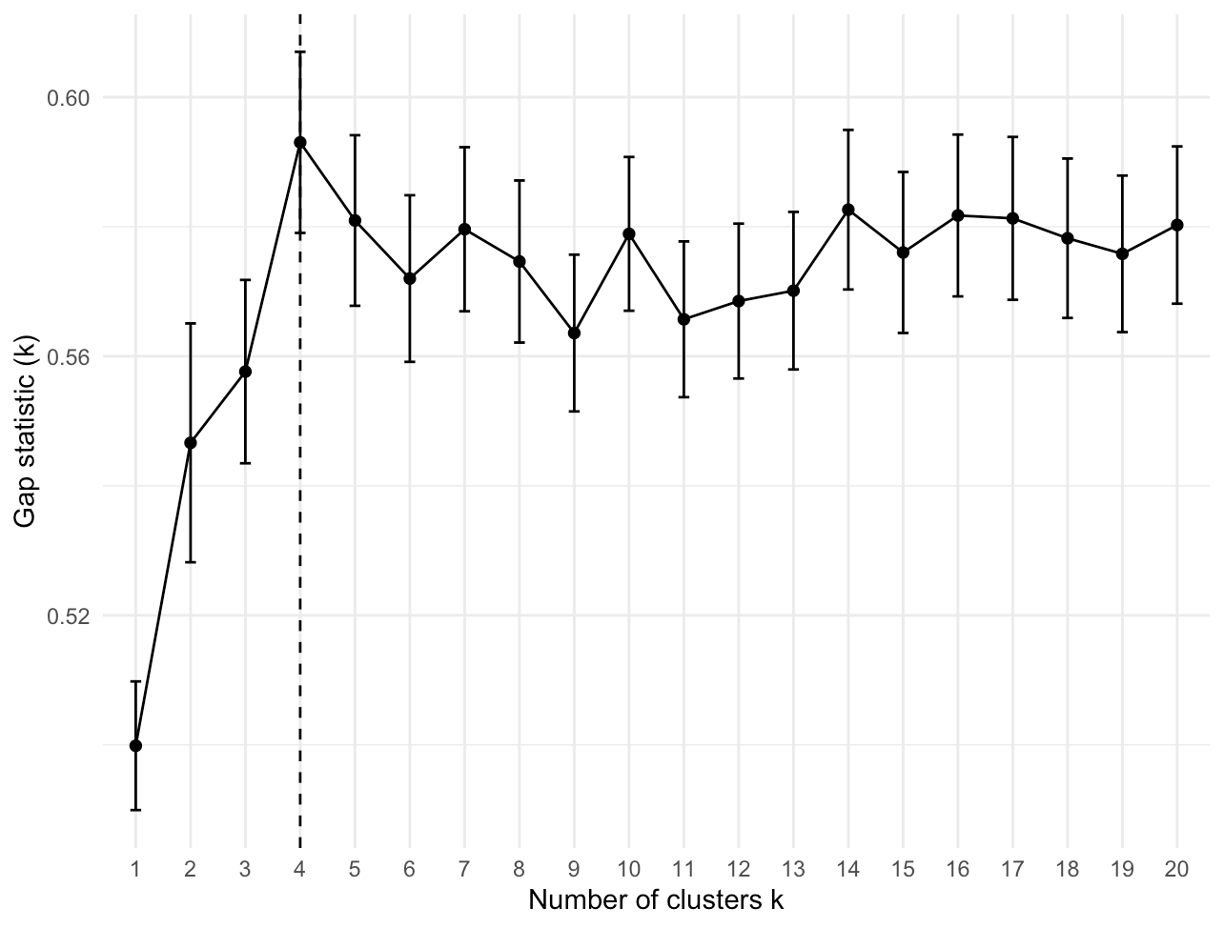

Supplement: Multimedia Appendix 4 [file pediatrics_v7i1e58757_app4.png]
